# Valuable Role of Neutrophil CD64 and Highly Sensitive CRP Biomarkers for Diagnostic, Monitoring, and Prognostic Evaluations of Sepsis Patients in Neonatal ICUs

**DOI:** 10.1155/2020/6214363

**Published:** 2020-08-07

**Authors:** Heba E. Hashem, Sherin A. El Masry, Amira M. Mokhtar, Eman A. Ismail, Noureldin M. Abdelaal

**Affiliations:** ^1^Clinical Pathology Department, Faculty of Medicine, Ain Shams University, Egypt; ^2^Neonatology Department, Faculty of Medicine, Ain Shams University, Egypt

## Abstract

**Background:**

Neonatal sepsis (NS) is a very critical medical situation associated with high morbidities and mortalities. There is an utmost need for a new tool helping in early diagnosis and proper management of sepsis neonates. Neutrophil CD64 (nCD64) shows a very promising value in this concerning issue.

**Aim:**

Evaluate the diagnostic, monitoring, and prognostic performances of nCD64 and highly sensitive CRP (hs-CRP) in NS as well as the possible best panel of biomarkers that can achieve the most desirable results.

**Methods:**

Patients were enrolled from three neonatal intensive care units (NICUs) (*n* = 121 patients) and classified according to their initial sepsis evaluation into three groups: disease control group (*n* = 30), proven sepsis group (*n* = 17), and clinical sepsis group (*n* = 74). Laboratory evaluation included hs-CRP, complete blood count (CBC), and blood culture in addition to nCD64 (done by flow cytometry technique). Besides the diagnostic evaluations, follow-up evaluations were done for 40 patients after five days from the first time; patients were reclassified according to their outcome into the improved sepsis neonates' group (*n* = 26) and sepsis neonates without improvement (*n* = 14).

**Results:**

Significant increase in nCD64 and hs-CRP results were present in sepsis groups compared to the disease controls (*P* < 0.001); nCD64 at 43% cutoff value could detect the presence of sepsis with 85.6% sensitivity and 93% specificity. Additionally, delta change percentage (dC%) between improved sepsis neonates and sepsis neonates without improvement showed a significant difference in the levels of both nCD64 (*P* < 0.001) and hs-CRP (*P* = 0.001).

**Conclusion:**

Besides the promising diagnostic performance documented by nCD64 which is higher than the other laboratory sepsis biomarkers used routinely in NICUs, nCD64 has a valuable role in sepsis patients' monitoring and prognostic evaluation. hs-CRP was moderate in its diagnostic and monitoring results being less than that achieved by nCD64. Combined measurement of nCD64% and hs-CRP gives better diagnostic and monitoring performance than that achieved by any of them alone.

## 1. Introduction

Sepsis remains a serious medical problem among the neonatal population, especially preterm infants [1, 2]. Its prevalence differs from one area to another depending on the presence of infection acquisition risk factors and infection control facilities [3, 4].

The highest incidence rate of neonatal sepsis (NS) is registered in Africa and Asia (23-38/1,000 live births) and the lowest in the U.S. and Australia (range 1.5-3.5/1000 live births) [5]. In South Asia, sub-Saharan Africa, and Latin America, the incidence is 7.6% with 9.8% annual case fatality accounting for 670000 deaths, and in fact, the worldwide deaths due to sepsis are double this number [6, 7].

Neonatal septicemia remains a diagnostic burden by showing minimal nonspecific initial manifestations with many diagnostic and monitoring pitfalls. In addition, the clinical course can be rapidly progressive and fatal if the suspected neonate is not managed properly at an early time [8, 9].

The blood culture remains the gold standard for sepsis diagnosis, even though its result is usually delayed for more than 48 hours. Additionally, there are false-positive results due to the impossibility of excluding contamination, besides its false-negative results which are frequently encountered in the neonatal population due to small unsatisfactory blood sample volume encountered in many circumstances in neonatal intensive care units (NICUs). The antibiotics administration before blood culture withdrawal adds another diagnostic obstacle increasing its false-negative results [10, 11].

Therefore, a more sensitive and specific diagnostic and prognostic tool is highly needed. Several hematologic markers have been investigated alone and in combination with other clinical and laboratory data with varying results [12, 13].

Despite the routine use of sepsis markers such as complete blood count (CBC) indices, C-reactive protein (CRP), and procalcitonin, there are many confounding factors, false positives, and false negatives which make them less ideal [14].

As a result, in the past few years, attention has been directed to other sepsis biomarkers including leukocyte cell surface antigens [8, 15].

Neutrophil CD64 (nCD64) is one of the most researchable markers in this aspect that have shown a particular promise in both early diagnosing and monitoring infections in both term and preterm newborns [10, 16, 17]. Even more, further studies postulated its good diagnostic performance for the discrimination between infectious and noninfectious systemic inflammatory response syndrome (SIRS) in the ICU setting [18].

nCD64 represents a membrane glycoprotein that mediates endocytosis, phagocytosis, antibody-dependent cellular toxicity (ADCC), cytokine release, and superoxide generation [19]. It is constitutively expressed on monocytes and macrophages; however, it is expressed at low concentration on nonactivated neutrophils but can be markedly upregulated at the onset of the sepsis process [20, 21].

The previous literature reported varying statistical results regarding nCD64 diagnostic performance in sepsis patients in addition to limited data concerning its monitoring and prognostic efficacy, so our aim in the present study was to evaluate nCD64 as a diagnostic, prognostic, and monitoring marker in NS comparable to the conventional laboratory methods and to determine the best panel of markers that can achieve the highest performance to be routinely applicable in neonatal ICUs.

## 2. Material and Methods

### 2.1. Study Design

The current study was a prospective hospital-based case-control study carried out at three Egyptian NICUs, over an eleven-month duration.

The study was approved by the Research Ethics Committee of Ain Shams University Hospitals, informed written consent was received from the parents of the enrolled neonates.

Subjected neonates were selected from NICU of Obstetric and Gynecological Hospital-Ain Shams University, NICU of Pediatric Hospital-Ain Shams University, and NICU of Pediatric Department-Ain Shams University specialized hospital.

### 2.2. Group Classification

One hundred and twenty-one neonates were consecutively enrolled and classified into three groups: group 1a—proven sepsis group (*n* = 17) which included those neonates with the clinical diagnosis of sepsis plus positive blood culture; group 1b—clinical sepsis group (*n* = 74) which included those neonates with the clinical diagnosis of sepsis but with negative blood culture.

In addition, thirty neonates were used as a disease control group (*n* = 30) (group 2). This group included those neonates with no signs of infection (the sepsis inclusion criteria were excluded in addition to negative CRP values throughout the study course); they are subjected to sampling for performing investigations of different diseases. This group of patients was age- and sex-matched with sepsis patients' groups.

Besides the diagnostic sepsis evaluations, 40 cases were subjected to further follow-up assessment after five days from their baseline evaluation. Patients were categorized into two groups according to their different outcomes: group 1—sepsis neonates without improvement (*n* = 14) which included neonates who were still in sepsis at the time of their follow-up evaluation without manifesting both clinical and laboratory improvement and group 2—improved sepsis neonates (*n* = 26) which included those who manifested clinically and laboratory improvement.

### 2.3. Patient Evaluation and Data Collection

Newborns enrolled in the study were subjected for thorough clinical examination, history taking (obtained directly from the parents and by accessing neonatal medical records), and samples of peripheral blood for the laboratory sepsis profile evaluation.

Demographic data were collected which included (1) gestational age (GA) classified into full-term (≥37 WK) and preterm neonates (<37 WK); (2) birth weight (BW) classified into very low BW (VLBW <1500 g), LBW (1500 g-2499 g), normal (2500-4000 g), and macrosomia (above 4000 g); and (3) sepsis risk factors including respiratory support, surgical procedures, and duration of hospitalization in addition to neonatal outcome (the outcome was evaluated after patients' follow-up till NICU discharge).

Preterm and term neonates showing clinical signs suggestive of early-onset sepsis (EOS) (within 72 hours of birth) or late-onset sepsis (LOS) (clinical deterioration after 72 hours) were eligible for the study.

### 2.4. Sepsis Definition

For sepsis patient's identification and selection, the presence of any antenatal risk factors (e.g. maternal Group B Streptococcus (GBS) colonization without adequate intrapartum prophylaxis, unknown maternal GBS status, maternal temperature, chorioamnionitis, preterm labor, or prolonged rupture of membranes) [22, 23], and the presence of at least 2 clinical and 2 laboratory criteria, the clinical criteria were [24] as follows:

1. Respiratory compromise: respiratory rate of >60 breaths per minute, cessation of respiration for ≥20 seconds, occurring at a rate of ≥2 times per hour, or pulse oximeter readings of ≤85%
2. Cardiovascular compromise: heart rate of <100 beats per minute, pallor, or hypotension
3. Metabolic changes: hypothermia (rectal temperature of <36°C), a body temperature of >38°C, feeding intolerance (increased gastric residuals of >50% of milk volume in ≥2 feedings within 24 hours), glucose instability (blood glucose level of <45 mg/dL or >125 mg/dL), or metabolic acidosis (pH < 7.25)
4. Neurologic changes: lethargy or decreased activity

The following are the laboratory criteria [25]:

- White blood cell (WBC) count < 5 or >20 × 10^9^ cells/L
- Immature to total neutrophil (*I* : *T*) ratio > 0.2
- Platelet count < 100 × 10^9^/L and CRP > 10 mg/L

The diagnosis was verified thereafter by positive blood culture.

### 2.5. Exclusion Criteria

Patients who had confirmed intrauterine viral infection (toxoplasmosis, rubella, cytomegalovirus, syphilis, and herpes), patients who recently undergone surgical intervention, and infants with neonatal asphyxia (Apgar score < 7 at 5 minutes) were excluded from the study.

### 2.6. Sample Collection and Measurements

Upon performing NS evaluation, the control group was subjected for one time of sampling at the baseline while two samples from sepsis group were obtained (one at the baseline and one after five days from the first assessment); the blood samples from sepsis group were withdrawn as early as the neonates were suspected clinically to have sepsis signs and symptoms.

Peripheral blood samples were collected on ethylenediaminetetraacetic acid (EDTA) for CBC and flow cytometric analysis of nCD64% and its mean fluorescence intensity nCD64 (MFI) while serum samples were obtained for the assessment of hs-CRP and chemistry profile (for the clinical judgment and follow-up purposes).

CBC samples were analyzed by the Coulter LH750 analyzer (Coulter Corporation, USA). Leishman stained blood smears were done for the estimation of immature/total neutrophil ratio (*I*/*T* ratio). hs-CRP was measured by dimension® clinical chemistry system.

Samples for blood culture were obtained; blood samples were withdrawn under complete aseptic conditions with a minimum of two mL blood inoculated into a BACTEC pediatric blood culture bottle.

The bottles showing positive signals were subjected to further subculture and processing. Isolates were then identified to the species level and tested for antimicrobial susceptibility by using the Vitek 2 system (BioMérieux, Marcy l'Etoile, France).

### 2.7. Flow Cytometric Analysis of nCD64

nCD64 expression was measured by flow cytometry technique as described by Choo et al. [24] using Leuko 64 assay (Leuko64 kit, Trillium Diagnostics, Scarborough, ME, USA). For sample preparation, peripheral blood samples on EDTA were processed and analyzed within two hours, up to 48 hours maximum of sample collection time.

Briefly, fifty *μ*L of well-mixed anticoagulated whole blood was incubated for ten minutes at room temperature with saturating amounts of fluorescein isothiocyanate-conjugated anti CD64 murine monoclonal antibody or isotype control (Leuko64 kit; Trillium Diagnostics), followed by ammonium chloride-based red cell lysis.

Samples were washed once and resuspended in 0.5 mL of phosphate-buffered saline with 0.1% bovine serum albumin. Flow cytometric analyses were performed using a Becton-Dickinson FACScan system to collect data on the logarithm of green fluorescein isothiocyanate, and linear right-angle side and forward scatter for a minimum of 5000 events was studied.

**Figure 1:**
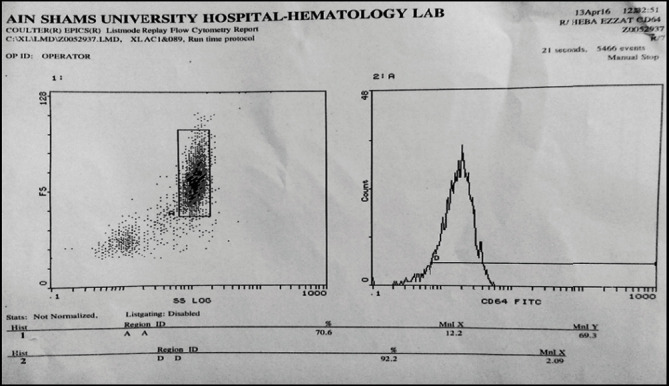
Flow cytometric result of nCD64 from a sepsis neonate enrolled in the study. CD64% was 92.2% and CD64 MFI was 2.09; this neonate, unfortunately, died later from severe septicemia.

Gating was done on the neutrophil cell population, based on its forward (cell size) and side scatter (granularity) properties, and the CD64 result was expressed as present and MFI units, and both were statically analyzed (Figure 1).

### 2.8. Statistical Analysis

It was performed by using SPSS statistical software package (V. 22.0). Data were expressed as the median and interquartile range for quantitative nonparametric number and percentage for presenting qualitative data.

A comparison between the three studied groups was performed by using the Kruskal-Wallis test while the comparison between every two independent groups was done by the Wilcoxon Rank Sum test; in addition, the correlation statistics (Spearman's correlation) for the possible associations between every two studied variables was conducted.

The diagnostic sensitivity, specificity, negative predictive value (NPV), positive predictive value (PPV), and efficacy (EFF) for each studied parameter were calculated. Receiver operating characteristic (ROC) curve and area under the curve (AUC) in addition to the calculated *Z* score and multiregression analysis were constructed to rank the independent factors in the current study.

## 3. Results

### 3.1. Patient Groups

Enrolled neonates were categorized according to their first sepsis evaluations into three groups: proven sepsis group (1a) (*n* = 17), clinical sepsis group (1b) (*n* = 74), and disease control group (2) (*n* = 30).

The proven sepsis and clinical sepsis groups were labeled as the sepsis group. The statistical analysis based mainly on the comparison between the combined septic groups (group 1a and group 1b; *n* = 91) and the disease control group (*n* = 30).

The disease control group included those neonates with no signs of infection, but they are subjected to sampling for performing investigations of different diseases including infants of a diabetic mother (IDM), prematurity, neonatal jaundice, hypoglycemic neonates, neonatal convulsion, a neonate with respiratory distress syndrome, and Hirschsprung disease with repeated attacks of hematemesis.

### 3.2. Patient Characteristics

The comparative statistics between the sepsis and the control groups regarding demographic, clinical, and laboratory data are illustrated in the Table 1.

Significant differences (*P* < 0.05) between both groups were documented regarding respiratory distress, the need for respiratory support, surgical interventions, the duration of hospital stay, and the number of deaths being higher in sepsis neonates compared to the disease controls.

Even though prematurity is a well-known sepsis risk factor, in the current study, no significant difference was documented between both the sepsis and control groups regarding gestational age (GA), birth weight (BW), or male gender (*P* > 0.05).

### 3.3. Blood Culture Results

The blood culture was positive in 46/91 of enrolled sepsis neonates (50% sensitivity) while its specificity was 90%.

**Figure 2:**
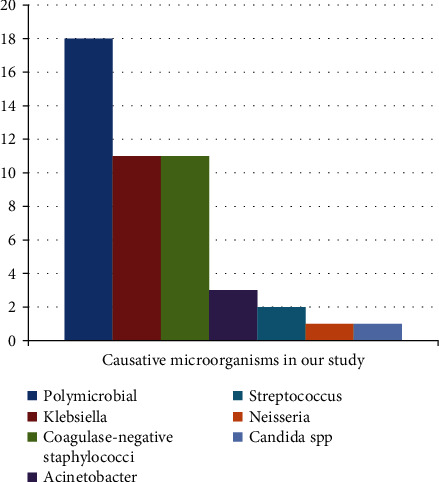
The distribution of the causative organisms.

The most common positive blood culture results revealed growth of more mono-microorganism; most of them were coagulase-negative *Staphylococcus* mixed with Gram-negative bacteria with 18 cases (40%), followed by coagulase-negative *Staphylococcus* with 11 cases (23.9%) and *Klebsiella species* 11 cases (23.9%); other bacterial species were less commonly isolated from our NICUs, e.g., *Acinetobacter*, *Streptococcus*, *Neisseria*, and *Candida* species (Figure 2).

**Figure 3:**
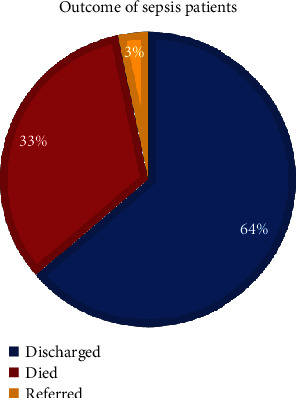
Outcome of sepsis patient in our study.

The outcome of subjected sepsis neonates was evaluated after patient follow-up until their NICU discharge. One-third of sepsis patients died from severe sepsis and its complications (Figure 3).

### 3.4. Laboratory Evaluation

In the current study, upon comparing the sepsis and disease control groups as regards the laboratory data (Table 1), highly significant differences in hs-CRP levels, *I*/*T* ratio, and nCD64 (both CD64% and CD64 MFI) were documented between both sepsis and controls being higher among sepsis patients while significant difference was documented by the platelet count between the groups being lower among sepsis neonates.

No significant difference was found regarding hemoglobin levels, total leukocyte count (TLC), or absolute neutrophil count (ANC).

**Figure 4:**
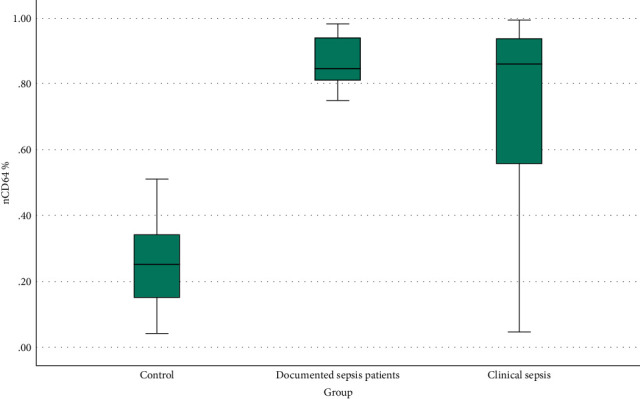
CD64 box blot between the three studied groups.

**Figure 5:**
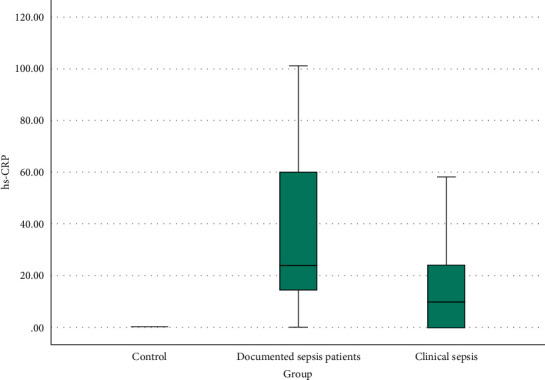
CRP box plot among the three studied groups.

Figures 4 and 5 represent CD64 and CRP box blots between the three studied groups.

CD64% significantly increased (*P* < 0.001) in both proven sepsis (median [IQR]: 0.856 [0.81-0.939]%) and clinical sepsis (median [IQR]: 0.86 [0.55025-0.93775]%) when compared to the disease controls (median [IQR]: 0.262 [0.15-0.365]%). But it cannot discriminate between both sepsis groups as no significant difference was found when proven sepsis and clinical sepsis were compared together (*P* = 0.398).

hs-CRP revealed a significant increase (*P* < 0.001) in both proven sepsis (median [IQR]: 24 [13.5-60] mg/dL) and clinical sepsis (median [IQR]: 10 [0-24] mg/dL) when compared to their matched controls (median [IQR]: 0 [0-0] mg/dL). Additionally, a statistically significant difference (*P* < 0.001) exists when proven sepsis and clinical sepsis were compared together (*P* = 0.009).

Within the sepsis group, 51.64% (47/91) of neonates had early-onset sepsis (EOS), and 48.35% (44/91) developed late-onset sepsis (LOS); the comparison was conducted between both groups regarding laboratory sepsis parameters (Table 2).

There was no statistically significant difference (*P* > 0.05) between both EOS and LOS groups regarding laboratory data except for hs-CRP that was lower in EOS than LOS (*P* = 0.003) and hemoglobin that were higher in EOS than LOS (*P* = 0.017).

Sepsis patients were furtherly subclassified according to NICU mortality into the nonsurvivor sepsis group (*n* = 30), and 2nd group included survivor sepsis patients (*n* = 61); the comparison was conducted between both categories of patients, and the results are illustrated Table 3.

Concerning the correlations between sepsis biomarkers, there were significant positive correlations between nCD64 and hs-CRP (rs = 0.248, *P* = 0.033) while nCD64 was negatively correlated with platelet count (rs = −0.298, *P* = 0.011), TLC (rs = −0.689, *P* = 0.002), and hemoglobin values (rs = −0.591, *P* = 0.012).

### 3.5. ROC Curve Analysis for Prediction of Sepsis Diagnosis

**Figure 6:**
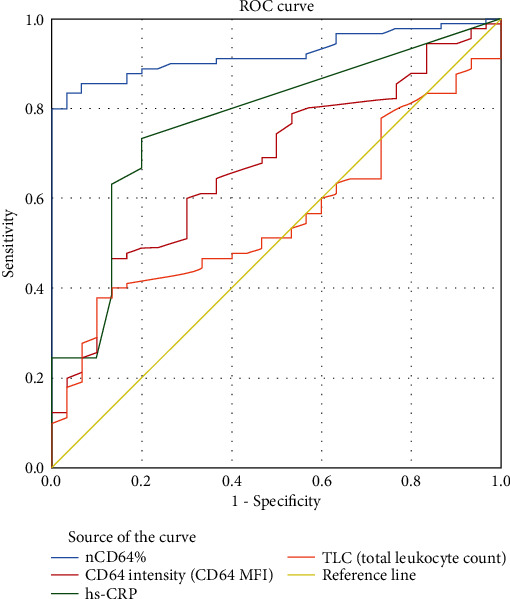
nCD64% achieved the highest diagnostic performance over the conventional sepsis parameters for discriminating patients with sepsis from those without.

ROC curve analysis showed that nCD64% at cutoff value 43% can achieve AUC = 0.922 with 85.6% sensitivity, 93% specificity, 68.3% NPV, 97.5% PPV, and 87.5% efficacy (Figure 6).

The simultaneous measurement of nCD64% and hs-CRP achieved the highest diagnostic performance with 91.2% sensitivity, 100% specificity, 100% PPV, 78.9% NPV, and 93.4% efficacy (Table 4).

### 3.6. Calculated *Z* Score

The *Z* score was calculated for both sepsis groups; the results demonstrated that nCD64% achieved a higher score followed by hs-CRP as a univariant diagnostic sepsis biomarker. The *Z* score mean values of nCD64% in both the proven sepsis and clinical sepsis groups were 4.6698 (1.185-5.79) and 3.73454 (-1.675-5.894), respectively, while the hs-CRP *Z* score mean values were 2.84013 (-0.423-8.118) and 1.34492 (-0.423-9.301), respectively. And so, the calculated *Z* score confirmed the previous statistical results.

### 3.7. Regression Analysis

The multiregression analysis was conducted in order to predict sepsis diagnosis; it included multiple models of different panels (Table 5). The best one included both nCD64% (*P* = <0.001) and CRP (*P* = 0.268) with the highest *F* ratio = 52.206.

### 3.8. Follow-Up Evaluations

By the follow-up of sepsis neonates, they had different clinical outcomes; they were reclassified into group 1 (sepsis neonates without improvement) (*n* = 14) and group 2 (improved sepsis neonates) (*n* = 26).

The comparison was conducted between both groups in terms of delta change (dC) percent (Table 6), the percentage change reflects how big the change is relative to the initial value, and it was calculated for each biomarker by using the following equation:

(1) $$\begin{array}{c} \frac{X\left(\text{final}\right)-X\left(\text{initial}\right)}{X\left(\text{initial}\right)}*100, \end{array}$$

where *X* stands for the sepsis parameter result.

The comparative statistics demonstrated that nCD64% achieved a superior result in the monitoring purpose (*P* < 0.0001) followed by hs-CRP (*P* = 0.001).

The *Z* score was calculated for both follow-up groups, and its results were confirmatory to what was previously achieved by delta change. The *Z* score mean values for nCD64% in both sepsis neonates without improvement and improved sepsis neonates' groups were 4.8248 (2.77-5.79) and 0.947(-1.89-3.75), respectively, while hs-CRP achieved lower values than CD64% being 3.4548 (-0.42-8.12) and 0.6984 (-0.42-7.69) in both groups, respectively.

**Figure 7:**
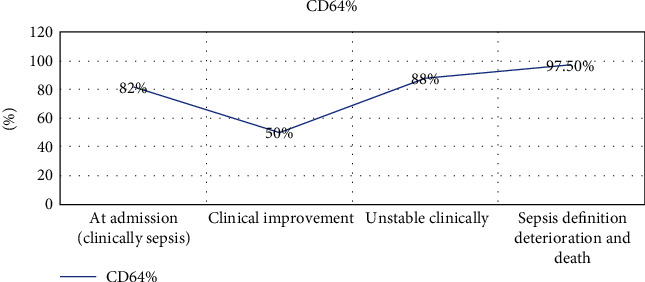
Multiple CD64% measurements in a sepsis patient receiving adequate antibiotic therapy then acquiring a nosocomial infection.

During the study course, we succeeded to follow five sepsis patients for more than three evaluations during their hospital stay; one patient was admitted for duodenal atresia surgical intervention; on admission, the neonate had clinical septicemia, and CD64% was 82% which decreased upon clinical improvement, reaching to 50%. Neonate had undergone surgical repair, and after two weeks later, CD64% levels raised again to 88% while the neonate was clinically unstable, after three days, CD64% was 97.5% where sepsis diagnosis was settled; the neonate died within a couple of days by fulminant neonatal septicemia proved by blood culture results which appeared after the neonatal death which was positive for *Klebsiella species* (Figure 7).

## 4. Discussion

Sepsis represents a serious medical problem especially among neonates [7, 25]. It is a major cause of the annual mortality in addition to the life-long morbidities among the survivors [6]. In the current study, the NICU mortality rate from neonatal septicemia was as high as 33% which comes in line with other studies results [12, 15, 26].

This study included 91 clinically septic neonates and 30 control neonates. Within the sepsis group, EOS was higher (51.64%) than LOS (48.35%). This observation was constant with previous studies [12, 27].

Blood culture is considered the gold standard for the diagnosis of NS. In the current study, 50% of neonates with clinically suspected sepsis had negative blood cultures. This rate is comparable to the rates reported in other developing African and Asian countries [28–30].

As regards the causative microorganisms, the most common blood culture results were positive for more mono-microorganism; most of them were coagulase-negative *Staphylococcus* mixed with Gram-negative bacteria (40%), followed by coagulase-negative *Staphylococcus* (23.9%) and *Klebsiella species* (23.9%). This comes in line with report of El-Din et al. [26] who reported the incidence of neonatal sepsis in both EOS and LOS was predominantly associated with Gram-positive cocci, specifically coagulase-negative *Staphylococcus* compared to Gram-negative and *Candida* spp. Similar findings were obtained in other studies in different countries [5, 31, 32].

On the contrary, El-Madbouly et al. [12] reported that K. pneumonia was the most frequent isolated organism from the blood of sepsis neonates (44.4%) which agreed with other studies [33]. This difference may be attributed to variation in local epidemiology and the microbial etiology of sepsis in addition to different care practices between medical centers.

In the current study, nCD64% achieved a highly significant increase in sepsis patients compared to the controls with a maximum efficacy of 87.5%, sensitivity of 85.6%, and specificity of 93%. These findings come in concordance with other studies results [20, 34–36].

The best cutoff of nCD64 was achieved at 43%; this cutoff is variable between the different researches including that of El Shimi et al. [35] who reported the best cutoff is 34.1%. These variations between the studies could be attributed to the difference in the number of the study population and the variation in their demographic and clinical characteristics in addition to the different used CD64 expression units [10, 12].

nCD64 was tested in most sepsis patients at very early stages; once sepsis was suspected even before overt signs of sepsis have been manifested, this indicates a valuable role of CD64 if used as a sepsis marker in early stages of sepsis process which is agreed by previous studies [10, 16, 20, 37].

Despite significantly higher values of CD64 in sepsis patients than the controls, it could not be discriminated between proven and clinical sepsis groups or between the EOS and LOS groups (*P* > 0.05) which comes in line with other results [12].

A significantly positive correlation between hs-CRP and nCD64% was documented; this could additionally reflect the clinical utility of CD64 in sepsis diagnosis which is agreed by El-Madbouly et al. and Behnes et al. [12, 38].

The current study proved that CRP is an effective biomarker for the differentiation between patients with sepsis and those without (*P* < 0.001) which is confirmed by some studies [10, 12, 39] while rejected by others [24]. These variations in the diagnostic performance of CRP between the studies could be attributed to the used CRP measuring technique, with the fact that immunoturbidimetric and nephelometric assay of CRP (hs-CRP) is more sensitive than the conventional technique measuring concentrations as low as 0.02 mg/dL [40].

Concerning CBC indices, the nonspecific nature of indices variations (TLC, ANC, and PLT) reflects their unsatisfactory sensitivity and specificity; as these parameters can be affected by many physiological and pathological conditions including the age of neonate, blood sampling method, mode of delivery, maternal hypertension, and even gender, so significant variations in their diagnostic performance between the different studies were reported [10, 22, 24].

In our study, the ROC curve analysis, in addition to the calculated *Z* score and multiregression analysis, proved that nCD64 achieved better results over the other conventional studied parameters including hs-CRP concerning sepsis diagnosis. This comes in line with the results by Aydin et al., El Shimi et al., and Elawady et al. [34, 35, 41].

On the opposite side, results of a meta-analysis conducted by Shi et al. [42] indicated that nCD64 expression alone in the diagnosis of neonatal sepsis should be treated with caution, and it is not a satisfactory marker for diagnosing neonatal sepsis with relatively low sensitivity and specificity despite relatively high ROC area, and it should be combined with the conventional hematological sepsis parameters or CRP to increase its diagnostic efficacy.

In our study, the combined measurement of nCD64% and hs-CRP achieved the highest diagnostic sensitivity and specificity of 91.2% and 100%, respectively. This result represents a promising diagnostic tool which is supported by Gilfillan et al., Choo et al., and El Shimi et al. [24, 35, 43].

For prognostic evaluation purposes, sepsis patients were reclassified according to NICU mortality into nonsurvivor sepsis (*n* = 30) and survivors sepsis patients (*n* = 61), the comparison was conducted between both categories, and the results showed significant differences between both groups regarding CD64% (*P* < 0.001), platelet count (*P* < 0.001), hs-CRP (*P* = 0.018), and CD64 MFI (*P* = 0.003). This valuable prognostic value of CD64 is supported by other results [44].

Additionally, 40 sepsis neonates in our study were monitored after 5 days from their first baseline evaluation; the results showed a valuable role of nCD64 in the monitoring aspect being better than hs-CRP and CBC indices; this was agreed by other studies [10, 45–47].

From our observations during the study course, we noticed the continuous variations of nCD64 results in context with the clinical changes in sepsis neonates. Also, we were able to follow five patients throughout their hospital stay for more than three successive evaluations, and their results were confirming the valuable role of nCD64 as a monitoring and prognostic sepsis marker.

Besides diagnostic, prognostic, and monitoring statistical results of nCD64%, it has some favorable technical characteristics. The expression of CD64 does not differ with age, as its expression only occurs upon cell activation [48] and is stable for more than 30 hours at room temperature [49]. Besides, the laboratory test for nCD64 is rapid (<60 minutes) and requires minimal blood volume (<100 *μ*L). In fact, in our study, no extra blood was obtained to calculate the nCD64% and nCD64 MFI, as CBC samples proved sufficient. Hence, it is practical to obtain nCD64 in a clinical setting. However, critical issues such as cost and clinical settings should be considered carefully before being routinely applicable.

For the current study, CD64 was measured only during the routine working hours using a FACScan system established at Flow Cytometry Unit-Clinical Pathology Department, which represents a major challenge of the clinical applicability of cell surface markers in the routine sepsis evaluation as the lack of a rapid and accurate point-of-care (POC) device that can perform its measurement from a minute blood sample. Recently, a robust biochip was designed by Hassan et al. [37]. This biochip can potentially be used for measuring nCD64 at the patient's bedside for continuous monitoring of his immune system in response to different therapeutic interventions at various stages of the disease, and this can facilitate greatly its availability in the routine daily sepsis evaluation in NICUs.

The second main obstacle for the routine application is the cost; in the current study, it costs approximately 60 L.E. to obtain one nCD64 test, which was more expensive than CRP's 40 L.E. However, changing the antibiotics prescribed, catching the false blood culture results, avoiding the performance of unnecessary organism typing, and antibiotic screening in addition to saving the cost of nursing care, bed charges, treatment complications, and antibiotic resistance as a whole will absolutely make the expense of performing this new biomarker which is, therefore, more than compensated for by the potential savings generated.

## 5. Conclusion

Neutrophil CD64 is a valuable diagnostic, prognostic, and monitoring biomarker for sepsis neonates. It achieved higher performance than the other conventional laboratory modalities studied. The diagnostic and monitoring capability of CD64 can be enhanced by using it in combination with CRP. Larger studies are needed to verify the nCD64 cutoff value before it could be widely incorporated in the routine daily practice.

### 5.1. Limitations of the Study

Limitations of this study must be addressed. Neonates with congenital malformations, chromosomal abnormalities, and surgical interventions were not excluded from the study, and this was intended to test the clinical application of sepsis biomarkers in the different heterogeneous groups of patients (which reflect the daily circumstances of our NICUs) before being routinely applicable.

## Figures and Tables

**Table 1:** Comparison between the sepsis and control groups as regards clinical and laboratory data.

Parameter	Sepsis group (*n* = 91)	Control group (*n* = 30)	*P* value
Preterm	54 (60.7%)	14 (47%)	0.180
LBW and VLBW	48 (53.2%)	10 (34.4%)	0.14
Male gender	47 (51.1%)	14 (47%)	0.674
Early onset sepsis	47 (51.64 %)	NA	NA
Respiratory distress	65 (71.4%)	2 (6.7%)	<0.001
Respiratory support	52 (57.1%)	0 (0%)	<0.001
Surgical interventions	20 (21.97%)	0 (0%)	<0.001
Nosocomial infection	38 (41.75%)	NA	NA
Deaths	30 (33%)	0 (0%)	0.01
DOH	24 (3-127)	5.5 (1–35)	<0.001
Hb (g/dL)	13.2 (8-21.4)	13 (6.4–23)	0.755
TLC (×10^9^/L)	15.3 (1-52)	14.4 (6.6–29)	0.281
ANC (×10^9^/L)	7.7 (0.6-40)	5 (1.6–14)	0.198
Platelet (×10^9^/L)	248 (15-620)	294 (70–587)	0.02
*I*/*T* ratio	0 (0- 0.9)	0 (0–0)	0.007
hs-CRP (mg/L)	10 (0-110.5)	0 (0–10)	<0.001
nCD64%	86 (46.7- 99.5)	26.2 (40.6–52.7)	<0.001
nCD64 MFI	1.84 (1.04- 4.62)	1.42 (1.05–3.56)	0.002

Values are presented as the median and IQR or number (%). *P* (probability value). DOH: duration of hospitalization; LOW: low birth weight; VLBW: very low birth weight; Hb: hemoglobin; TLC: total leukocyte count; ANC: absolute neutrophil count; *I*/*T* ratio: immature/total neutrophil ratio; hs-CRP: highly sensitive CRP; nCD64%: neutrophil CD64%; nCD64MFI: nCD64 mean fluorescence intensity; NA: not available.

**Table 2:** Comparison between EOS and LOS.

	Early-onset sepsis group (EOS)	Late-onset sepsis group (LOS)	*Z*	*P*
	Median (IQR)	Median (IQR)		
Hb (g/dL)	13.5 (11.875-15.275)	11.8 (10.1-14.2)	-2.385	0.017
TLC (×10^9^/L)	13.55 (9.05-23.325)	15.9 (10.1-20.2)	-0.624	0.533
ANC (×10^9^/L)	8 (3.35-13.675)	6.7 (4.25-11.4)	-0.306	0.76
Platelet (×10^9^/L)	218.5 (123-287.25)	220.5 (67.75-342.75)	-0.351	0.725
hs-CRP (mg/L)	9 (0-24)	24 (6.5-48)	-2.986	0.003
CD64%	0.8725 (0.61575-0.94125)	0.8185 (0.54275-0.9275)	-0.997	0.319
CD64 MFI	2.125 (1.6025-2.7075)	1.81 (1.32-2.5575)	-1.505	0.132

Hb: hemoglobin; TLC: total leukocyte count; ANC: absolute neutrophil count; PLT: platelet; hs-CRP: highly sensitive CRP; nCD64%: neutrophil CD64%; nCD64MFI: nCD64 mean fluorescence intensity.

**Table 3:** Comparison between nonsurvivor sepsis patients and survivor sepsis patients.

	Nonsurvivor sepsis group	Survivor sepsis group	*Z*	*P*
	Median (IQR)	Median (IQR)		
GA (weeks)	34 (32.75-37)	36 (34-38)	-2.192	0.028
BW (grams)	2700 (2000-3200)	2580 (1900-3212.5)	-0.076	0.939
Hb (g/dL)	11.15 (9.8-12)	13.1 (10.675-14.8)	-3.507	<0.001
TLC (×10^9^/L)	10.65 (8.15-17.8)	14.6 (9.65-19.95)	-2.17	0.03
ANC (×10^9^/L)	6.5 (2.4-12.4)	7 (4.4875-12.225)	-1.11	0.267
Platelet (×10^9^/L)	90 (29-168)	240 (158-321.75)	-5.252	<0.001
hs-CRP(mg/L)	24 (9.75-96)	12 (6-37)	-2.368	0.018
CD64%	94.1 (83-97.6)	81.85 (60.075-89.375)	-4.213	<0.001
CD64 MFI	2.15 (1.76-3)	1.82 (1.44-2.41)	-2.958	0.003

GA: gestational age; BW: birth weight; Hb: hemoglobin; TLC: total leukocyte count; ANC: absolute neutrophil count; PLT: platelet; hs-CRP: highly sensitive CRP; nCD64%: neutrophil CD64%; nCD64MFI: nCD64 mean fluorescence intensity.

**Table 4:** The diagnostic performance of studied sepsis biomarkers.

	Cutoff value	Specificity (%)	Sensitivity(%)	NPV (%)	PPV (%)	Eff (%)	AUC
CD64%	0.43	93%	85.6%	68.3%	97.5%	87.5%	0.922
hs-CRP (mg/L)	3.0	80%	73.3%	50%	91.7%	75%	0.772
CD64 MFI	1.445	50.0%	73.3%	38.5%	81.5%	68.0%	0.675
TLC (×10^9^/L)	14.7	53%	51.1%	26.7%	76.7%	51.6%	0.561
ANC (×10^9^/L)	5	52.9%	67.5%	26.5%	86.7%	64.9%	0.684
*I*/*T* ratio	<0.2	100.0%	25.7%	49.0%	100.0%	56.7%	0.779
Blood culture	+ve results	90.0%	50.0%	37.5%	93.8%	60.0%	—
CD64% & hs-CRP (mg/L)	0.43 + 3.0	100%	91.2%	78.9%	100%	93.4%	0.988

Eff.: efficacy; NPV: negative predictive value; PPV: positive predicted value.

**Table 5:** Multiregression analysis.

	Odd's ratio (95% CI for odd's ratio)	Significance	*F* ratio
Model 1:			
TLC_1	0.833 (0.534–1.300)	0.421	
ANC	1.501 (0.797–2.828)	0.208	
I_T_Ratio	3.214E+42 (0.000–0.000)	0.994	
Plt_1	1.004 (0.991–1.017)	0.540	
hs-CRP_1	4.801 (0.000–2.190*E* + 213)	0.995	
CD64%_1	105376.110 (0.001–18623361548434.200)	0.233	
			8.974
Model 2:			
hs-CRP_1	1.023 (0.983-1.065)	0.268	
CD64%_1	1699.272 (81.061-35621.535)	0.0001	
			52.206

**Table 6:** Delta changes (dC) percent for both sepsis neonates without improvement (group 1) and improved sepsis neonates (group 2).

	Sepsis neonates without improvement (Gr1)	Improved sepsis neonates (Gr2)	*Z*	*P*
HB_dC	-6.629 (-13.105–17.85)	-9.43 (-18.246–0.224)	-0.992	0.321
TLC_dC	16.277 (-27.688–45.433)	-5.153 (-35.398–47.281)	-0.851	0.395
ANC_dC	27.174 (5.357–404.03)	-38.235 (-0.56911–0.30303)	-2.415	0.016
Plt_dC	-2.41 (-73.62–13.876)	17.925 (-0.0551–1.07197)	-2.056	0.04
CRP_dC	50 (-25–640.833)	-80 (-100 to -50)	-3.427	0.001
CD64%_dC	0.691 (-4.806–19.154)	-47.423 (-71.136–34.091)	-5.031	<0.0001
CD64 MFI_dC	0.942 (-28.648–47.023)	-26.337 (-47.075–10.106)	-1.65	0.09

Values are presented as the median and IQR.

## Data Availability

The (original research) data used to support the findings of this study have been deposited in the 4TU.Centre for Research Data repository (https://researchdata.4tu.nl/en/home/). The authors declare that the data underlying the findings of this research are publicly available.
